# Experiences of Obstetrician-Gynecologists Providing Pregnancy Care After *Dobbs*

**DOI:** 10.1001/jamanetworkopen.2025.2498

**Published:** 2025-03-31

**Authors:** Abigail S. Cutler, Corinne M. Hale, Eliza Bennett, Laura Jacques, Jenny Higgins

**Affiliations:** 1 Department of Obstetrics and Gynecology, University of Wisconsin-Madison; 2 Department of Anthropology, University of Wisconsin-Madison; 3 Collaborative for Reproductive Equity (CORE), University of Wisconsin-Madison

## Abstract

**Importance:**

Following the *Dobbs v Jackson Women’s Health Organization* (*Dobbs*) decision in June 2022, which overturned the federal right to abortion, Wisconsin physicians faced the threat of an 1849 state law widely interpreted to criminalize provision of abortion except in life-saving emergencies. Physicians and their institutions were left to interpret whether and how they could treat and/or refer certain pregnant patients.

**Objective:**

To document how the post-*Dobbs* legal landscape shaped Wisconsin obstetrician-gynecologists’ (OB-GYNs’) ability to provide health care to patients facing pregnancy-related risks and complications, with particular attention to the mediating role of health care institutions.

**Design, Setting, and Participants:**

In this qualitative study, 21 OB-GYNs were recruited between June 2022 and December 2023 from rural and urban areas with varying hospital affiliations, scopes of practice, and individual demographics to participate in semistructured, remote interviews.

**Exposure:**

An 1849 abortion law that suspended abortion care in Wisconsin between June 2022 and December 2023.

**Main Outcomes and Measures:**

Physicians’ perceptions of (1) how the legal landscape shaped management of pregnancy and related complications, and (2) how institutional-level factors contributed to physicians’ experiences caring for pregnant patients following *Dobbs*.

**Results:**

This study included 21 OB-GYNs (mean [SD] age, 43 [5.88] years; 16 [76.2%] female; 5 [23.8%] non-White and 16 [76.2%] White) who practiced obstetrics in Wisconsin. OB-GYNs described how the threat of criminalization following *Dobbs* was detrimental to physicians’ ability to provide pregnancy care. Absence of legal clarity surrounding the 1849 law led to confusion and wide variations in institutional comfort and clinical practice, which resulted in substandard, delayed, and fragmented patient care. Overwhelmingly, the threat of criminalization after *Dobbs* exacerbated barriers for physicians providing comprehensive pregnancy care and patients seeking it.

**Conclusions and Relevance:**

In this qualitative study of OB-GYNs practicing in an abortion-restrictive state, threat of criminalization in post-*Dobbs* Wisconsin resulted in uncertainty and confusion for OB-GYNs and worse care for pregnant patients. The absence of clear guidance and support from institutional and health care system leadership emerged as a particularly salient missed opportunity. These experiences, which contribute to a critical evidence base on the harms of abortion restrictions, are relevant to states facing similar bans that criminalize or restrict health care.

## Key Points

**Question:**

How did the fall of *Roe v. Wade* change how Wisconsin obstetrician-gynecologists (OB-GYNs) cared for patients facing pregnancy-related risks and complications?

**Findings:**

In this qualitative study describing the experiences of 21 Wisconsin OB-GYNs practicing under the threat of criminalization after *Dobbs v Jackson Women’s Health Organization* (*Dobbs*), participants described uncertainty and confusion for physicians, lack of clear guidance from hospitals and health systems, and worse care for pregnant patients.

**Meaning:**

These findings suggest that after *Dobbs,* the threat of a state abortion ban was detrimental to Wisconsin OB-GYNs’ ability to provide standard of care to patients facing pregnancy complications.

## Teaser

This qualitative study evaluates how the legal landscape after *Dobbs v Jackson Women’s Health Organization *shaped Wisconsin obstetrician-gynecologists’ ability to provide health care to patients facing pregnancy-related risks and complications.

## Introduction

Abortion has been heavily restricted for decades. Evidence demonstrates that state-level abortion restrictions have long impeded access to a wide range of obstetric services beyond abortion, including treatment for miscarriage and ectopic pregnancies.^1,2,3,4^ Following the US Supreme Court *Dobbs v Jackson Women’s Health Organization* (*Dobbs*) decision on June 24, 2022, many states further banned or severely restricted abortion. A number of such laws, either trigged by the fall of *Roe* or passed after, contain criminal penalties for physicians.^5,6^ It is more important than ever to document the impact of abortion restrictions on pregnancy care.

Following the *Dobbs* decision, an 1849 Wisconsin state law widely interpreted to be an abortion ban posed criminal penalties to any physician who provided an abortion for any reason except to “save the life of the mother.”^7^ Because of preexisting restrictions limiting hospital-based abortion care, freestanding abortion clinics had long provided the vast majority of abortions in Wisconsin, and these clinics immediately ceased providing abortion services.^8^ Hospitals and outpatient clinics across the state followed suit, leaving physicians without clarity about when it was legal to provide standard care for patients facing complications of pregnancy. Pregnant Wisconsinites were forced to navigate unfamiliar and complex systems to obtain abortion services as well as care for pregnancy complications—often across state lines.^9,10^

Termination of pregnancy may be offered or recommended due to numerous pregnancy complications, according to widely accepted standards of care.^11,12,13,14,15,16,17^ For 13 months following *Dobbs*, nearly no abortions were performed in Wisconsin. In September 2023, a state circuit court issued a preliminary decision that the 1849 statute does not apply to voluntary abortions, and Planned Parenthood of Wisconsin resumed abortion services in 2 locations.^18,19^ Following a final ruling issued by the same court in December 2023, some Wisconsin hospitals and practices also resumed abortion services in accordance with pre*-Dobbs* state laws.^8,20^ As of February 2025, some hospitals and health systems still cite abortion-related legal uncertainty as reason for withholding certain services for pregnant patients.

We sought to document how the post-*Dobbs* legal landscape shaped the abilities of Wisconsin obstetrician-gynecologists (OB-GYNs) to provide health care to patients facing pregnancy-related risks and complications between June 2022 and December 2023. We also explored the role of health care systems in mediating these outcomes, as such systems and institutions remain underexamined influences on the relationship between abortion restrictions and pregnancy care provision.

## Methods

This qualitative study followed the Standards for Reporting Qualitative Research (SRQR) reporting guidelines. The study received exempt status from the University of Wisconsin-Madison institutional review board (IRB) following limited and expedited IRB review.^21^ OB-GYNs provided verbal consent to participate in the study.

### Study Population

OB-GYNs who cared for patients facing pregnancy complications in the hospital or outpatient setting and had practiced in Wisconsin at least 1 year before and 1 year following *Dobbs* were eligible to participate. Trainees (medical students, residents, and fellows) were ineligible.

### Recruitment

We created a theoretical sampling framework intended to achieve diversity across age, gender, race and ethnicity, practice location (urban vs rural), and scope and setting of clinical practice. Investigators identified and recruited prospective participants through informal and formal professional networks, snowball sampling, and direct contact via email or text message. After 21 interviews, the researcher team agreed theoretical sufficiency had been reached.^22^

### Data Collection

We developed and piloted a semistructured interview guide (see eAppendix in Supplement 1) based on a review of the relevant literature, expert input, and our study objectives. We explored (1) professional background and scope and location of clinical practice; (2) post-*Dobbs* changes in management of common pregnancy complications; (3) institutional practices, policies, and support; (4) perceived impact of criminal ban on professional and personal life; and (5) perceived threats to OB-GYN workforce. We piloted the interview guide with 2 OB-GYNs (L.J. and E.B.) who were part of the study team before formal data collection. We collected participant demographics during interviews via self-report and/or through publicly available professional online profiles. Interviews were conducted by either C.M.H. or A.S.C. over Zoom and lasted 45 to 60 minutes. Audio recordings were professionally transcribed and deidentified. Participants received a $100 gift card upon interview completion.

### Data Analysis

Two study team members (C.M.H. and A.S.C.) coded transcripts in Dedoose version 9.0.107 (Dedoose) using a thematic qualitative approach^23^; we iteratively compiled, consolidated, and refined all codes through discussion until reaching consensus. Additional study team members (J.H., E.B., and L.J.) offered input on coding discrepancies as needed. C.M.H. and A.S.C. then engaged with relevant coding reports to create the basis of the themes and subthemes detailed in the Results section, seeking input from J.H., E.B., and L.J. throughout their thematic development refinement.

## Results

The final sample included 21 OB-GYNs (mean [SD] age, 43 [5.88] years; 16 [76.2%] female; 5 [23.8%] Asian, Black, and Hispanic; and 16 [76.2%] White) from diverse practice settings (Table).^24^ Seven participants had completed a subspecialty fellowship in either maternal-fetal medicine or complex family planning. Our analysis revealed 2 interconnected themes that captured the experiences of Wisconsin OB-GYNs following the *Dobbs* decision: absence of legal clarity and perceived impact on the provision of care. Across interviews and themes, health care institutions played a decisive role in shaping physician experiences and perceived impact on patients.

**Table.** Obstetrician-Gynecologist (OB-GYN) Participant Characteristics by Self-Report

Characteristic	Participants, No. (%) (N = 21)
Gender	
Male	5 (23.8)
Female	16 (76.2)
Age group, y	
<35	0
35-45	16 (76.2)
46-55	4 (19.1)
≥56	1 (4.7)
Race and ethnicity	
White	16 (76.2)
Asian, Black and Hispanic^a^	5 (23.8)
Scope of practice^b^	
General OB-GYN	18 (85.7)
Subspecialty fellowship	7 (33.3)
Institution affiliation/type^b^	
Religious	5 (23.8)
Academic	10 (47.6)
Community-based	11 (52.4)
Population density	
Urban	17 (81.0)
Rural	4 (19.0)

^a^ Asian, Black, and Hispanic participants were combined to protect participant anonymity.

^b^ Categories are not mutually exclusive; includes self-reported and publicly-available data.

### Absence of Legal Clarity

Participants described the 1849 Wisconsin statute as “vague,” and “uninterpretable,” which created inherent conflict between providing evidence-based clinical care and adhering to the law both at the individual and institutional level. Two subthemes emerged.

#### Imprecise Wording of Law and Unclear Institutional Guidance

Most participants described uncertainty regarding how to provide standard clinical care within the bounds of a statute that failed to define when intervening to “save the life of the mother” was legally permissible. Physicians noted that this exception was “not really interpretable,” “not a medical term,” and “difficult to define.” Several described how confusion and uncertainty impacted clinical care:

The available wording about what is life-threatening to the mother is very subjective and leaves room for a lot of debate about how life-threatening it is. If I’m offering somebody an abortion, and they have a 5% chance of dying if they don’t have an abortion, is that good enough for me to offer that and be legally protected?

Not knowing how to translate the 1849 legal language into clinical practice, participants relied on their institutional leaders (hospital or practice administrators, department chairs, and legal teams) to provide guidance, which varied widely across hospitals and health systems. In interviews, some participants referenced institutional guidance that allowed them to offer standard of care (including pregnancy termination) for certain pregnancy complications, while other participants cited institutional guidance prohibiting them from providing pregnancy termination because of the 1849 law, even for the same pregnancy complication. Several participants lamented a lack of expert guidance about what care physicians were “allowed” to provide:

I wish the state would have provided our administrators, our chief medical officer, and our risk management or quality department with more guidance about how to support providers and patients. Because...we don’t always know how to interpret legal language. We didn’t really get guidance about how to execute ourselves, either administratively or clinically, in the new landscape.

Not all participants trusted the institutional guidance they did receive, leading to variations in care even within institutions, and exacerbating confusion over how to legally and safely provide pregnancy care:

While we did make these decisions [about when to intervene] as an institution and as a department...not everybody in the department felt comfortable proceeding with offering care because they were worried about the legal risk for themselves.

Some participants perceived their institutions as prioritizing the health system over their patients and clinicians:

I felt disappointed that some of the legal counsel that I was receiving may have been grounded more in concern for protecting the health system rather than protecting the patient’s best interests or even mine as a physician who wanted to be able to be providing this care because it’s health care.

#### Feelings of Legal Vulnerability and Internal Conflict

Whether or not their institutions offered clear legal guidance, many participants described having to make their own determinations of how much personal legal risk they would be willing to incur to “do the right thing” for their patients. Several indicated that their malpractice insurance would not cover criminal liability posed by the 1849 statute, which rendered physicians vulnerable:

I’ve done everything I can to make sure that I’m abiding by the law. But...if I were charged with a felony charge for the 1849 ban, my employer would want nothing to do with me; they would wash their hands of me as fast as possible and say they had nothing to do with [the abortion]. You’re very much on your own to defend yourself.

Participants described deep conflict between upholding their obligations to patients and jeopardizing their personal safety, well-being, and freedom. One physician described weighing the risks of doing the “right thing”:

I had to look at myself in the mirror and say, “Well, [providing an abortion] is fundamentally the right thing for this patient, and I can’t envision how anyone would say otherwise. So, I’m going to do it, and the consequences will be what they are.” But I think about my whole family, my whole career...I think of everything that would be, poof, gone.

Physicians experienced legal vulnerability differently. Several participants indicated their identity as a parent weighed heavily in calculations over how much legal risk they were willing to incur:

As much as I am here for my patients and want to do everything I can to give them the care they need, at the end of the day, I’m still a human. I’m a mom. I have this life that I’ve worked really hard for, and I want to provide for my children. I can’t afford to go to prison to do this.

One physician felt especially vulnerable because they had always been an “outspoken abortion provider” and thus worried about a “target on [their] back.” Another participant described how their racial identity shaped their decision-making around whether to participate in some aspects of pregnancy care following *Dobbs*:

In Wisconsin and across America, certain groups like African Americans receive harsher criminal sentences. I was concerned that there would be disparities in incarceration, where some would go free and others would spend the full time in jail.

### Substandard, Delayed, and Fragmented Care After *Dobbs*

The threat of criminalization and the uninterpretable language of the statute had implications for many clinical scenarios, including pregnancies affected by early pregnancy problems (miscarriage or ectopic pregnancy), second trimester complications (preterm premature rupture of membranes [PPROM] or placental abruption), chronic health conditions, and fetal anomalies. Physicians were most immediately concerned with how to legally and safely manage previable PPROM. Certain Wisconsin institutions ultimately allowed physicians to offer standard management options (expectant management, induction of labor, or dilation and evacuation), but several participants shared that their institutions had deemed intervention too risky. One participant described a patient with previable PPROM 2 weeks following *Dobbs*:

Because of [the *Dobbs*] ruling, despite how early she was and how poor the outcomes would be in that circumstance, we were no longer able to offer an induction or surgical procedure in that circumstance until the patient’s life became in danger. Before we could intervene on her behalf, she either needed to basically become infected, septic, or she needed to start to hemorrhage, as long as there were fetal heart tones.

This physician continued to describe the implications of expectant management, which refers to monitoring a patient’s condition without providing immediate medical or surgical intervention:

The patient ended up getting infected. [Only] then we were allowed to proceed with the induction. She did go on to deliver vaginally a stillborn infant. However, she became so sick that she was admitted to the [intensive care unit (ICU)] with concerns for sepsis. And so, what should have been less than a 48-hour stay turned into a 5-day hospital admission, including ICU care.

Many physicians stressed that the law rendered clinicians unable to consider the individual needs of their patients, including those with cancer or chronic health conditions that could worsen because of pregnancy. One physician described caring for a patient whose early pregnancy posed significant risk to her underlying health, but not sufficient risk to warrant abortion care under the 1849 statute, according to institutional leadership:

The patient had decided she didn’t want to be pregnant [because she] had a lot of medical comorbidities that would have made an ongoing pregnancy very dangerous. However, none of those comorbidities or medical problems included previable rupture of membranes, preeclampsia, hemorrhage. She was deemed by a committee of people to not meet the threshold by which we could confidently intervene and still feel like we were following the law.

The 1849 statute also caused many physicians to be exceedingly cautious in their care and require more diagnostic testing for suspected but not yet definitive pregnancy failure—regardless of a patient’s own preferences regarding management. One physician described:

My partners [and I] have all experienced patients coming in with a very clear nonviable pregnancy. However, because of the implications of the law, we have to have them come back for multiple images and labs to quote “confirm” this before we can intervene, which results in a lot more emotional and physical distress for these patients, potentially increasing their risk for other complications like hemorrhage or infection.

Participants noted an increase in transfers of care between health care systems and hospitals following *Dobbs,* which disrupted continuity of care, contributed to treatment delays, and resulted in clinical morbidity (such as sepsis and hemorrhage). Due to differing interpretations of the statutory language across institutions even within the same state, larger referral hospitals found themselves on the receiving end of patient transfers from other institutions whose physicians did not feel comfortable offering—or were not permitted to offer—intervention for previable PPROM:

[PPROM] was a major source of problems […] we got a lot of transfers, people that got really sick waiting for care because they couldn’t intervene earlier or thought they couldn’t intervene earlier.

The same physician described the experience of caring for one such patient:

A patient had experienced previable PPROM. In her home community, she was informed that they couldn’t offer anything while the baby was still alive or while she didn’t have an obvious infection. Ultimately, she came to our institution to get care.

Several participants reported that their health care institutions took exceedingly conservative positions regarding services related even remotely to abortion, including pregnancy options counseling. This phenomenon was most salient at institutions with particularly restrictive or unclear policies regarding abortion even before *Dobbs.* Although the 1849 statute did not restrict counseling about abortion, conservative readings of the law led to constraints above and beyond the provision of abortion care itself:

[I’ve been told] I can’t document anything for them [if we talk about abortion], and I can’t provide them with any written information in that case.... The guidance that we were given in our system was that we were not allowed to provide any written information about obtaining terminations.

Among the patients forced to travel out of state for abortion care were those with (even fatal) fetal anomalies, unless they were experiencing a pregnancy complication deemed immediately life-threatening. Physicians described how crossing state lines was most difficult for those patients who already struggled to access any type of health care:

There’s...all the logistical hurdles that patients need to overcome to access the care: […] gas, money, transportation, lodging once they’re there, childcare because most patients who have abortions are already parents, missed time from work. […] All of these logistical barriers fall hardest on people who already have trouble accessing health care, patients who are already marginalized in our health care system—Black, Brown, Indigenous patients, patients with low-incomes or low resources, patients who are in rural settings. I’ve seen a lot of inability to access care at all because of where they live. These patients are disproportionately disadvantaged and burdened.

## Discussion

In this qualitative study, OB-GYNs from across the state and from a range of health care settings described far-reaching outcomes of the *Dobbs* decision on the provision of pregnancy-related health care in Wisconsin. Specifically, threat of criminalization posed by an 1849 state law banning abortion led to uncertainty and confusion for physicians caring for pregnant patients and substandard, delayed, and fragmented care. The absence of clear guidance and support from institutions and health care systems was a particularly relevant and harmful factor that shaped the experiences of physicians responsible for providing pregnancy care as well as the perceived experiences of patients seeking it.

Findings are consistent with research in other abortion-restricted states where criminal bans have caused legal uncertainty and confusion, impeded the provision of evidence-based pregnancy care, and jeopardized the well-being and professional integrity of the physician workforce.^24,25,26,27,28,29,30,31,32^ Our study specifically examined the mediating role of health care systems and institutions in the relationship between abortion restrictions and physician experience and patient care. Leading medical and professional organizations insist that to ensure high-quality, evidence-based, patient-centered health care, the patient-physician relationship must be free of political interference.^33^ However, Wisconsin OB-GYNs—not unlike physicians in other states where abortion care is heavily restricted by state law or institutional policies—felt that conservative and inconsistent interpretation of laws too often protected institutions at the expense of patients and physicians. Furthermore, the lack of consistent legal interpretation across institutions within the same state severely compounded confusion for physicians and harms for patients. Participants in our study frequently cited insufficient or disappointing guidance from institutional leadership—hospital and practice administrators, executives, and legal teams—which rendered them unable to provide timely, evidence-based care to patients facing pregnancy complications.

Our findings suggest the necessity for health systems facing statewide and/or institutional abortion restrictions to provide clear clinical guidance, meaningful legal resources, and robust referral processes to protect the well-being of both patients and clinicians. In the face of ambiguous state laws such as Wisconsin’s 1849 statute, consistency in legal interpretation across institutions is particularly important to reduce confusion among physicians and patients alike.

To our knowledge, this study is the first to document the myriad harms caused by the threat of a criminal abortion ban in Wisconsin following the fall of *Roe.* Study strengths include the participation of OB-GYNs from a diversity of state regions and clinical settings, including religiously affiliated hospitals that restricted abortion care before *Dobbs*. Data collection occurred before a judicial ruling in December 2023 that returned abortion access to some Wisconsin hospitals and health systems according to pre*-Dobbs* restrictions, which reduces risk of retrospective bias. At a time when maternal mortality is unacceptably high,^34^ maternity care deserts are growing in number,^35^ and rates of moral distress among physicians are increasing,^36,37,38^ it is critical to understand the impact of abortion restrictions on broader reproductive and obstetrical health care—including both patients’ ability to receive needed care and clinicians’ ability to freely practice evidence-based medicine and provide the best possible health care to their patients.

### Limitations

Our study is limited by potential selection bias; physicians in our sample may have stronger views on abortion restrictions than those who declined or did not answer our invitations to participate. Further research is needed on the ongoing impacts of *Dobbs* on states’ non–OB-GYN workforce and on patients themselves, particularly in light of an ever-evolving legal landscape that, while serving to restore abortion access to some parts of the state for some patients, has not provided the needed clarity for all institutions to resume abortion services or offer them in the first place.

## Conclusion

In this qualitative study of OB-GYNs practicing in an abortion-restrictive state, threat of criminalization in post-*Dobbs* Wisconsin led to uncertainty and confusion for OB-GYNs and substandard care for pregnant patients. Findings from this study elevate the critical role of health care systems and institutions in this relationship, which have the potential to be either barriers or facilitators in the provision of health care, even while functioning under the same restrictions. Our study findings also contribute to a critical, state-specific evidence base on the highly visible as well as less obvious harms of abortion restrictions at all levels, which remain ever relevant to settings that criminalize, obstruct, or limit the provision of pregnancy-related health care.

## Notes

Supplement 1. **eAppendix. **Clinician Interview Guide

Supplement 2.
Data Sharing Statement
