# Error in Introduction

**DOI:** 10.1001/jamanetworkopen.2025.15018

**Published:** 2025-05-05

In the Original Investigation titled “Experiences of Obstetrician-Gynecologists Providing Pregnancy Care After *Dobbs*,”^1^ published March 31, 2025, there was a typographical error in the Introduction. This article has been corrected.^1^
