# Supplementary material for: Experiences of Obstetrician-Gynecologists Providing Pregnancy Care After Dobbs
Source: JAMA Netw Open. 2025 Mar 31;8(3):e252498. doi: 10.1001/jamanetworkopen.2025.2498 (PMC11959436; doi:10.1001/jamanetworkopen.2025.2498)
Supplement: Supplement 1. — eAppendix. Clinician Interview Guide [file jamanetwopen-e252498-s001.pdf]

Supplemental Online Content

Cutler AS, Hale CM, Bennett E, Jacques L, Higgins L. Experiences of obstetrician-gynecologists providing pregnancy care in a post-*Dobbs* Wisconsin. *JAMA Netw Open*. 2025;8(3):e252498. doi:10.1001/jamanetworkopen.2025.2498

eAppendix. Clinician Interview Guide

This supplemental material has been provided by the authors to give readers additional information about their work.

© 2025 Cutler AS et al. *JAMA Network Open*.

eAppendix. Clinician Interview Guide

Study Title: When Pregnancies Become Complicated: Consequences of Dobbs for Patients and Ob-Gyn Physicians in Wisconsin

Principal Investigator: Abigail Cutler, MD, MPH

Hello, my name is [STATE NAME] from the University of Wisconsin-Madison.

Thank you for agreeing to participate in this interview and share your experience.

Before we begin, I want to briefly describe the research study to you and confirm your interest in participating. Please feel free to ask any questions along the way.

As a reminder, the purpose of this study is to explore and document how the current legal landscape has changed how Wisconsin Ob-Gyns provide care for patients who face pregnancy-related risks and complications. We are also interested in examining the institutional and system-level factors that have shaped physicians' experiences providing this care.

Your participation will be confidential and coded, which means you will be assigned a random ID number to be linked to your interview in lieu of your name. You will be sent a $100 gift card via email for completing the interview. Would you still like to participate? [Wait for confirmation.]

This interview is optional, and you can tell me if you want to stop at any time or you want to skip any question. Do you have any questions about this or any time constraints I should be aware of? [Wait for response and answer questions as they arise.]

Were you able to review the information sheet we sent to you? Do you have any questions regarding the study details or questions for the research team? Do you consent to participating in the interview as detailed in the information sheet? [Wait for confirmation].

I'd also like your permission to audio record this interview. You may ask me to stop recording at any time. Do I have your permission to record? [Wait for confirmation then request that both interviewer and participant turn off their Zoom video.]

Please try to avoid mentioning names or any details that could be used to directly identify yourself or others as well as medical facilities where you work or where your patients may receive care. If you do happen to mention any identifying information, we will remove it during transcription. Do you have any questions about that? [Wait for response]

© 2025 Cutler AS et al. *JAMA Network Open*.

Great. We're ready to begin.

Workplace & Training

First, I am going to ask you questions regarding your workplace, professional background, and training.

1. Regarding your workplace:
    a. In what type of setting is your practice? Inpatient (hospital-based, including L&D, ER GYN) vs outpatient (clinic-based) vs mixed?
    b. What is the scope of your clinical practice? (Probe: Outpatient OB? Gynecology? Labor and delivery?)
    c. Has your scope ever included providing abortion care here in Wisconsin?
        i. If yes: please describe the abortion training you received.
        ii. If no, did your practice include referring patients for abortion care?
    d. What is your practice type? Academic (University-affiliated? Teaching hospital?), non-academic, private practice, hospital-employed, or community-based?
    e. What size is your practice or institution? (Probe: Are you part of a tertiary care center?)
    f. Do you practice in a rural or urban area? What region of Wisconsin (Southern, Eastern (Southeast and Northeast), Northwest (Western and Northern))?
    g. How would you describe the characteristics of your patient population? What is the general income level, racial/ethnic make-up, medical status (e.g., high risk pregnant people) of your patient population?
    h. Does your hospital or health system have any religious affiliation? What about any religiously-informed constraints on care offered (abortion, sterilization, IVF, ectopic pregnancy)?

2. Prior to the *Dobbs* decision in June 2022, did your hospital or health system offer any abortion services? Did your institution place any non-religious constraints on what kind of abortion care (under what circumstances; to whom) could be provided?

Part I: Providing Care in Post-Dobbs Wisconsin

Next, I will be asking you questions about your experience providing pregnancy-related care in Wisconsin.

3. Are you familiar with the 1849 Wisconsin statute on abortion? Can you describe it for me?

© 2025 Cutler AS et al. *JAMA Network Open*.

4. Following the *Dobbs* decision, can you describe how your ability or willingness to provide pregnancy care changed? Has it evolved at all from where it initially started?

5. I'd like to hear more about the way your clinical practice has changed because of the current legal landscape. Has the legal landscape changed the way you provide care (or help patients obtain care) for:
    a. Patients with unwanted pregnancy?
    b. Patients with diagnosed ectopic pregnancy?
    c. Patients with suspected or known miscarriage?
    d. Patients with pregnancies of unknown location?
    e. Patients with suspected or known molar pregnancy?
    f. Patients with high-risk pregnancies due to underlying maternal health conditions?
    g. Patients affected by fetal anomalies?
    h. Patients whose pregnancies resulted from rape or incest?
    i. Patients whose pregnancies are affected by unexpected obstetric complications (eg. PPROM, hemorrhage, preeclampsia, diabetes)

6. Thinking about patients you've taken care of since *Dobbs* who have experienced complications in pregnancy, can you think of any instances in which the care you or others provided differed from the usual standard, due to the need comply with the post-*Dobbs* legal landscape?

7. Do you discuss the 1849 statute and its implications with your patients? In what context(s) do these discussions arise?

8. Do you currently discuss the option of abortion with patients who seek it? Do you feel comfortable referring patients for abortion care or helping to facilitate it?

9. Beyond its direct impact on patient care, how do you see or imagine Wisconsin's abortion restrictions impacting the lives of your patients outside the clinical setting? Do you notice that the impact is bigger or different on some patients more so than on others? If so, who and in what ways?

10. Do you think more patients are taking reproductive care into their own hands under the current circumstances? If so, how?

© 2025 Cutler AS et al. *JAMA Network Open*.

11. As you know, some abortion access returned to Wisconsin earlier this fall. Has that been enough? In your mind, what else needs to change in order for your patients to have access to all the pregnancy care they need?

Part II: Institutional Factors Shaping Physician Experiences

Next, we're going to go over institutional factors that may be shaping physician experiences during this time.

12. Thinking back to June 2022 when the *Dobbs* decision was announced, how did your institution respond? Who formulated the institution's response? (Probe: clinical staff, administrators, legal counsel) Were there any committees formed to formulate a response, create new workflows or clinical protocols, enact new restrictions on care?

13. Does your institution's interpretation of the 1849 statute and its application to the way you provide pregnancy-related health care differ at all from your own interpretation of the law?

14. Following *Dobbs*, did your institution implement any other new guidelines or restrictions, beyond clinical care? (Probe: New rules on engaging with media; medical education/training)

15. Has your institution implemented any support systems specifically for patients seeking care for pregnancy complications?

16. Did your perception of your employer change after the *Dobbs* decision?

17. Have your workplace relationships changed at all since or surrounding the *Dobbs* decision? What about relationships with your patients?

Part III: Exploring Support for Physicians Caring for Pregnant Patients

Now, we're going to talk more in depth about the support you've had (or wish you'd had) as a physician who cares for pregnant patients in a post-Dobbs Wisconsin.

© 2025 Cutler AS et al. *JAMA Network Open*.

18. Following *Dobbs*, has your institution put in place any supports to help physicians better care for patients in this legal landscape? Are there types of support you wish you and/or your colleagues were receiving that would help you better navigate the post-*Dobbs* legal landscape – professionally or personally?

19. Can you speak to whether/how the threat of a felony charge posed by the 1849 statute has impacted you personally? (Probe: Is prosecution under this statute something you've worried about? Have you had conversations with your family about the possibility of criminalization related to your work?)

20. Have your professional plans changed since the *Dobbs* decision? (Probe: have you ever questioned your decision to continue practicing medicine in Wisconsin?) What advice would you give to a professional colleague who was thinking about moving to Wisconsin to practice Ob-Gyn?

21. Do you think the post-Dobbs legal landscape in Wisconsin has impacted the ability to recruit and retain Ob-Gyn residents and physicians?

22. We are nearing the end of our interview. Is there anything else you would like our research team to know about your experience or the current state of pregnancy care in Wisconsin?

Demographics

Before we wrap up, I'd like to gather some demographic information about you.

a. How old are you?
b. How do you identify your gender?
c. How do you identify your race or ethnicity?

Thank you again for taking the time to participate in this interview. We will destroy your contact information now that we have completed the interview. If you are interested in learning about the findings from this study, please reach out to us at (608) 262-9960 or email WIpostdobbsstudy_obgyn@wisc.edu.

© 2025 Cutler AS et al. *JAMA Network Open*.
