# Supplementary material for: Experiences of Obstetrician-Gynecologists Providing Pregnancy Care After Dobbs
Source: JAMA Netw Open. 2025 Mar 31;8(3):e252498. doi: 10.1001/jamanetworkopen.2025.2498 (PMC11959436; doi:10.1001/jamanetworkopen.2025.2498)
Supplement: Supplement 2. — Data Sharing Statement [file jamanetwopen-e252498-s002.pdf]

Data Sharing Statement

Cutler. Experiences of Obstetrician-Gynecologists Providing Pregnancy Care After Dobbs. *JAMA Netw Open*. Published March 31, 2025. doi:10.1001/jamanetworkopen.2025.2498

Data
Data available: No
